# Zinc Transporter 3 (ZnT3) in the Enteric Nervous System of the Porcine Ileum in Physiological Conditions and during Experimental Inflammation

**DOI:** 10.3390/ijms18020338

**Published:** 2017-02-07

**Authors:** Sławomir Gonkowski, Maciej Rowniak, Joanna Wojtkiewicz

**Affiliations:** 1 Department of Clinical Physiology, Faculty of Veterinary Medicine, Oczapowskiego 13, University of Warmia and Mazury, 10-718 Olsztyn, Poland; 2 Department of Comparative Anatomy, Faculty of Biology, Plac Łódzki 3, University of Warmia and Mazury, 10-727 Olsztyn, Poland; maciek@matman.uwm.edu.pl; 3 Department of Pathophysiology, Faculty of Medical Sciences, Warszawska 30, University of Warmia and Mazury, 10-082 Olsztyn, Poland; asiawoj@uwm.edu.pl; 4 Laboratory for Regenerative Medicine, Faculty of Medical Sciences, University of Warmia and Mazury, Olsztyn, 10-082 Olsztyn, Poland; 5 Foundation for Nerve Cells Regeneration, Warszawska 30, 10-082 Olsztyn, Poland

**Keywords:** zinc transporter 3 (ZnT3), enteric nervous system (ENS), inflammation, pigs

## Abstract

Zinc transporter 3 (ZnT3) is a member of the solute-linked carrier 30 (SLC 30) zinc transporter family. It is closely linked to the nervous system, where it takes part in the transport of zinc ions from the cytoplasm to the synaptic vesicles. ZnT3 has also been observed in the enteric nervous system (ENS), but its reactions in response to pathological factors remain unknown. This study, based on the triple immunofluorescence technique, describes changes in ZnT3-like immunoreactive (ZnT3-LI) enteric neurons in the porcine ileum, caused by chemically-induced inflammation. The inflammatory process led to a clear increase in the percentage of neurons immunoreactive to ZnT3 in all “kinds” of intramural enteric plexuses, i.e., myenteric (MP), outer submucous (OSP) and inner submucous (ISP) plexuses. Moreover, a wide range of other active substances was noted in ZnT3-LI neurons under physiological and pathological conditions, and changes in neurochemical characterisation of ZnT3^+^ cells in response to inflammation depended on the “kind” of enteric plexus. The obtained results show that ZnT3 is present in the ENS in a relatively numerous and diversified neuronal population, not only in physiological conditions, but also during inflammation. The reasons for the observed changes are not clear; they may be connected with the functions of zinc ions and their homeostasis disturbances in pathological processes. On the other hand, they may be due to adaptive and/or neuroprotective processes within the pathologically altered gastrointestinal tract.

## 1. Introduction

The enteric nervous system (ENS) is localised in the wall of the gastrointestinal (GI) tract and takes part in all regulatory processes connected with digestive actions, such as intestinal motility and excretion [1]. It is characterised by significant independence from the central nervous system, as well as complex conformation. The ENS is made up of millions of neurons grouped in intramural ganglionated plexuses interconnected with a very dense network of nerves. The number and form of these plexuses clearly depend both on the animal species and the fragment of the GI tract [1,2,3]. In the porcine intestine, the ENS is built of three intramural plexuses: the myenteric plexus (MP)—located between the longitudinal and circular muscle layers, the outer submucous plexus (OSP)—in the inner side of the circular muscle layer, and the inner submucous plexus (ISP)—between the muscularis mucosa and lamina propria [4]. Enteric neurons vary in terms of their conformation, functions, electrophysiological properties and neurochemical coding [2]. Besides acetylcholine, which is the main transmitter in enteric neurons [5,6], several dozen other neuronal active substances have been described within the ENS [2,3]. The most important of these include, among others, vasoactive intestinal peptide (VIP), galanin (GAL), neuronal isoform of nitric oxide synthase (nNOS) and substance P (SP) [7,8,9]. Most of the abovementioned factors act as neuromediators and/or neuromodulators, but the functions of some neuronal substances described in the ENS remain unknown. One of these is zinc transporter 3 (ZnT3) [10].

ZnT3 is one of the solute-linked carrier 30 (SLC 30) protein family of zinc transporters, which in mammals consists of 10 members (ZnT1 to ZnT10) [11]. These molecules allow zinc ions to permeate from the cytoplasm to the intercellular space, as well as intracellular organelles [12]. From among the ZnT family of zinc transporters, only ZnT3 is closely linked to neuronal cells, where it is responsible for transport of zinc ions from the cytoplasm to synaptic vesicles, and thereby may influence neuronal conduction [12]. ZnT3 has been described in the brain, spinal cord and autonomic peripheral nervous system [13,14,15]. Within the central nervous system, this protein may be responsible for both sensory conduction and secretory activity and is also considered to be a marker of zinc-enriched nerves (ZEN), which have an inhibitory function [16]. Furthermore, it is known that ZnT3 can take part in some pathological processes within the central nervous system, such as epilepsy, mechanical damage or ischemia [15,17,18].

In contrast to the brain and spinal cord, knowledge about the distribution and functions of ZnT3 in the peripheral autonomic nervous system is very sparse [14]. This is particularly visible with reference to the ENS. Admittedly, ZnT3 has been described in the human descending colon [10], as well as in the porcine duodenum and jejunum [19,20], but some aspects of the functions of this peptide within the GI tract, especially during pathological states, remain within the realm of conjecture.

The roles of ZnT3 are probably closely linked to the functions of zinc in a living organism. This metal is known to be a very important factor, as it is a component of various enzymatic systems and takes part in the stabilisation of cellular membranes, DNA synthesis, cell division and the correct functioning of the immune system [21]. Relatively high levels of zinc have been described in the central nervous system, where this metal is indispensable to normal brain development and neuronal functioning [22]. Moreover, “free pool” reactive zinc ions can act as neurotransmitters and/or neuromodulators [23]. Zinc also plays an important role within the GI tract, where first of all it stimulates the absorption of ions in enterocytes by modulating intracellular cAMP concentration, and therefore it is used as a drug to decrease the severity and duration of diarrhoea [24]. Moreover, zinc as a component of zinc finger E-box-binding homeobox (Zeb) 2 protein takes part in the formation, migration and specification of cells in the neural crest, from which the ENS arises, and is also necessary for the correct functioning of enteric neurons [25].

On the other hand, it is relatively well known that the ENS regulates the functions of the intestinal mucosa, which is the first barrier against various pathological factors [1]. Therefore, enteric neurons may undergo changes caused by adaptive and/or neuroprotective processes during many intestinal and extra-intestinal diseases, and these changes mainly manifest themselves in modifications of neuronal chemical coding [25,26,27,28,29].

The aim of the present study was to investigate the changes in ZnT3-like immunoreactive (ZnT3-LI) enteric neurons in the porcine ileum during experimental chemically induced inflammation. The choice of the pig as the experimental animal during the present study was determined by the fact that this species more and more often appears to be an optimal animal model for pathological processes in the human organism, due to similarities in anatomical, histological and physiological properties between humans and pigs [30,31,32].

## 2. Results

In the present study, neurons immunoreactive to ZnT3 were observed within the porcine ileal enteric nervous system, both under physiological conditions and during inflammation (Table 1). All ZnT3-positive cells noted in the ganglionated plexuses of the ENS were also immunoreactive to protein gene product 9.5 (PGP 9.5—used as a panneronal marker). Moreover, during the present investigation, ZnT3-LI cells were noted outside of the enteric plexuses. These cells were scattered in various parts of the intestinal wall and were not PGP 9.5-positive.

### 2.1. ZnT3 in the ENS under Physiological Conditions

**Figure 1:**
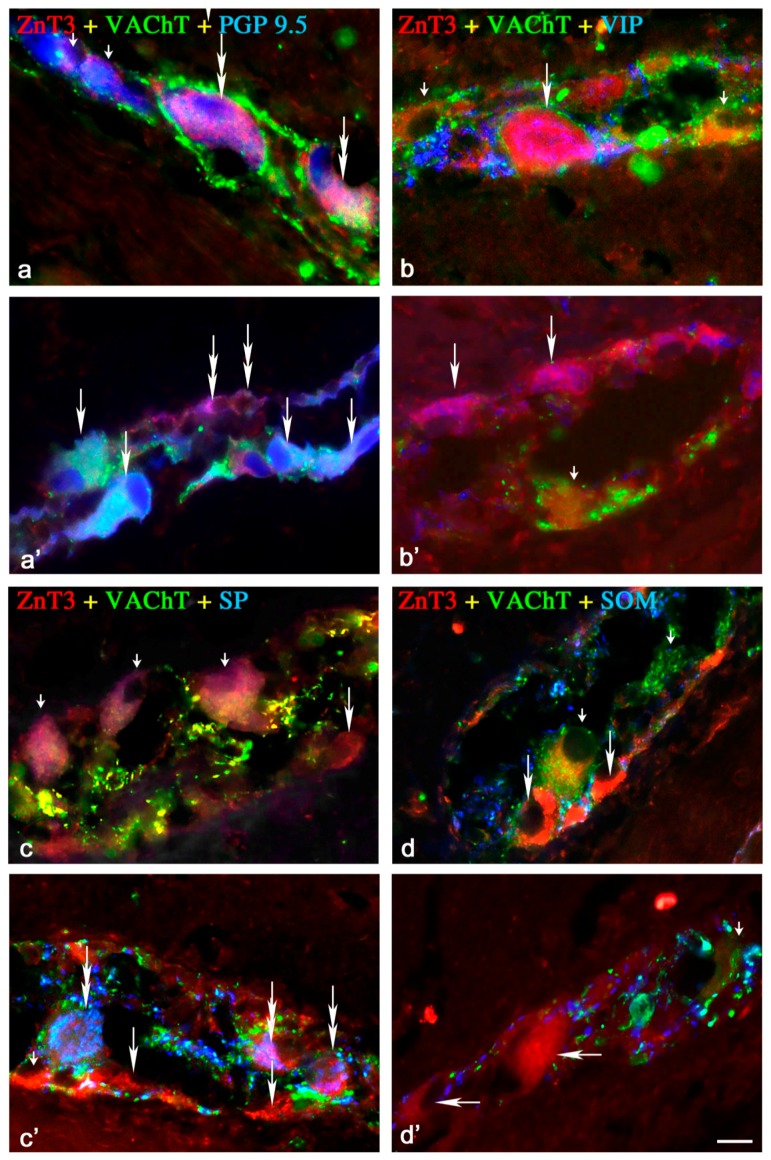
Representative images of ZnT3^+^ neurons located in the myenteric plexus (MP) of the porcine ileum: myenteric ganglia. All images are composites of merged images taken separately from blue, red and green fluorescent channels. Control (C) group: (**a**) ZnT3^+^/VAChT^+^/PGP9.5^+^ neurons are indicated with double-headed arrows, ZnT3^−^/VAChT^−^/PGP9.5^+^ neurons are indicated with small arrows; (**b**) ZnT3^+^/VAChT^+^/VIP^+^ neurons are indicated with an arrow, ZnT3^+^/VAChT^+^/VIP^−^ neurons are indicated with small arrows; (**c**) ZnT3^+^/VAChT^+^/SP^−^ neurons are indicated with small arrows; ZnT3^+^/VAChT^−^/SP^−^ neurons are is indicated with arrows; (**d**) ZnT3^+^/VAChT^+^/SOM^−^ neuron is indicated with a small arrow; ZnT3^+^/VAChT^−^/SOM^−^ neurons are indicated with arrows. Inflammatory (I) group (**a’**) ZnT3^+^/VAChT^+^/PGP9.5^+^ neurons are indicated with arrows, ZnT3^+^/VAChT^−^/PGP9.5^+^ neurons are indicated with double-headed arrows; (**b’**) ZnT3^+^/VAChT^−^/VIP^+^ neurons are indicated with arrows; ZnT3^+^/VAChT^−^/VIP^−^ neurons are indicated with a small arrow; (**c’**) ZnT3^+^/VAChT^+^/SP^−^ neuron is indicated with a small arrow; ZnT3^+^/VAChT^−^/SP^+^ neurons are indicated with double-headed arrows; ZnT3^+^/VAChT^−^/SP^−^ neurons are indicated with arrows; Control group (**d’**) ZnT3^+^/VAChT^+^/SOM^−^ neuron is indicated with a small arrow; ZnT3^+^/VAChT^−^/SOM^−^ neurons are indicated with arrows. Scale bar 25 µm.

**Figure 2:**
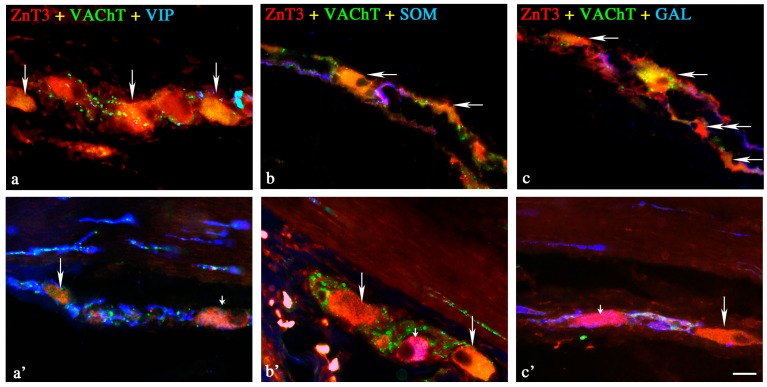
Representative images of ZnT3^+^ neurons located in the outer submucous plexus (OSP) of the porcine ileum. All images are composites of merged images taken separately from blue, red and green fluorescent channels. Control (C) group: (**a**) ZnT3^+^/VAChT^+^/VIP^−^ neurons are indicated with arrows; (**b**) ZnT3^+^/VAChT^+^/SOM^−^ neurons are indicated with arrows; (**c**) ZnT3^+^/VAChT^+^/GAL^−^ neurons are indicated with arrows; ZnT3^+^/VAChT^−^/GAL^−^ neuron is indicated with a double-headed arrow. Inflammatory (I) group: (**a’**) ZnT3^+^/VAChT^+^/VIP^−^ neuron is indicated with an arrow; ZnT3^+^/VAChT^−^/VIP^+^ neuron is indicated with a small arrow; (**b’**) ZnT3^+^/VAChT^−^/SOM^+^ neuron is indicated with a small arrow; ZnT3^+^/VAChT^+^/VIP^−^ neurons are indicated with arrows; (**c’**) ZnT3^+^/VAChT^−^/GAL^+^ neuron is indicated with a small arrow; ZnT3^+^/VAChT^+^/GAL^−^ neuron is indicated with an arrow. Scale bar 25 µm.

**Figure 3:**
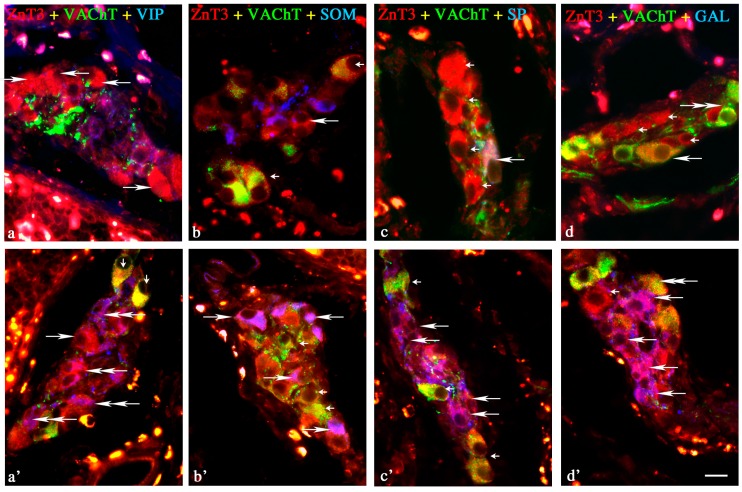
Representative images of ZnT3+ neurons located in the inner submucous plexus (ISP) of porcine ileum. All images are composites of merged images taken separately from blue, red and green fluorescent channels. Control (C) group: (**a**) ZnT3^+^/VAChT^−^/VIP^−^ neurons are indicated with arrows; I group (**b**) ZnT3^+^/VAChT^+^/SOM^−^ neurons are indicated with small arrows; ZnT3^+^/VAChT^−^/SOM^−^ neuron is indicated with an arrow; (**c**) ZnT3^+^/VAChT^−^/SP^−^ neurons are indicated with small arrows; ZnT3^+^/VAChT^+^/SP^+^ neuron is indicated with an arrow; (**d**) ZnT3^+^/VAChT^−^/GAL^−^ neurons are indicated with small arrows; ZnT3^+^/VAChT^+^/GAL^−^ neuron is indicated with an arrow; ZnT3^−^/VAChT^+^/GAL^−^ neuron is indicated with a double-headed arrow; Inflammatory (I) group: (**a’**) ZnT3^+^/VAChT^+^/VIP^−^ neurons are indicated with small arrows; ZnT3^+^/VAChT^−^/VIP^+^ neurons are indicated with double-headed arrows; ZnT3^+^/VAChT^−^/VIP^−^ neuron is indicated with an arrow; (**b’**) ZnT3^+^/VAChT^−^/SOM^+^ neurons are indicated with arrows; ZnT3^+^/VAChT^+^/SOM^−^ neurons are indicated with small arrows; (**c’**) ZnT3^+^/VAChT^−^/SP^+^ neurons are indicated with arrows; ZnT3^+^/VAChT^+^/SP^−^ neurons are indicated with small arrows; (**d’**) ZnT3^+^/VAChT^+^/GAL^−^ neuron is indicated with a double-headed arrow; ZnT3^+^/VAChT^−^/GAL^−^ neuron is indicated with a small arrow; ZnT3^+^/VAChT^−^/GAL^+^ neurons are indicated with arrows. Scale bar 25 µm.

Under physiological conditions, the percentage of these neurons was relatively high, levelised in all “kinds” of plexuses, and amounted to 42.3% ± 4.7% in the MP (Figure 1), 43.5% ± 6.8% in the OSP (Figure 2) and 48.6% in the ISP (Figure 3). In individual enteric ganglion, most often three or more ZnT3-LI cells were noted (Figure 2c), but ganglia with one or two such perikarya were also observed (Figure 1a). There are no differences between particular “kinds” of plexuses in the distribution of ZnT3^+^ cells in individual enteric ganglia.

#### 2.1.1. Neurochemical Characterisation of ZnT3^+^ Cholinergic Enteric Neurons

A wide range of neuronal active substances was investigated in ZnT3-positive enteric neurons under physiological conditions (Table 1). A significant percentage of cells were immunoreactive to ZnT3, regardless of whether the “kind” of enteric plexus was cholinergic (positive to vesicular acetylcholine transporter—VAChT) (Figure 1a,c,d, Figure 2a–c and Figure 3b–d). These neurons accounted for 85.0% ± 5.8%, 82.0% ± 5.9% and 90.0% ± 7.2% of all ZnT3^+^ perikarya in the MP, OSP and ISP, respectively. Neurochemical coding of cholinergic neurons immunoreactive to ZnT3 was variable in different “kinds” of enteric plexuses. Some ZnT3^+^/VAChT^+^ neuronal cells in the MP and OSP were also immunoreactive to somatostatin (SOM), VIP and SP, but the degree of co-localisation of ZnT3 with the abovementioned particular substances was slight and did not exceed 7.5% (Table 1).

A completely different situation was observed in the ISP. In this plexus, a much higher percentage of cholinergic ZnT3-LI neurons were immunoreactive to SOM, VIP and/or SP. These values amounted to 24.1% ± 1.2%, 33.0% ± 3.6% and 55.1% ± 1.3%, respectively (Table 1). Moreover, 65.2% ± 1.2% of cholinergic ZnT3^+^ cells in the ISP were also immunoreactive to GAL, contrary to MP and OSP, where ZnT3^+^/VAChT^+^/GAL^+^ neurons were not observed at all. During the present study, immunoreactivity to nNOS, leu-enkephalin (LENK), neuropeptide Y (NPY) and calcitonin gene-related peptide (CGRP) was not observed in cholinergic ZnT3^+^ neurons in any enteric plexus.

#### 2.1.2. Neurochemical Characterisation of ZnT3^+^ Non-Cholinergic Enteric Neurons

Non-cholinergic neurons in the MP amounted to 15.0% ± 5.6% of all ZnT3^+^ cells (Figure 1a,c,d). In the OSP and ISP, these values stood at 18.0% ± 5.9% and 10.0% ± 7.2%, respectively (Figure 2c and Figure 3b). The degree of co-localisation of ZnT3 with other substances studied in non-cholinergic cells clearly depended on the “kind” of plexus (Table 1). The most visible differences were observed in the event of nNOS and GAL. In the MP, 17.1% ± 1.1% of non-cholinergic ZnT3^+^ cells were also nNOS-positive, whereas in the OSP, this value amounted to only 7.4% ± 1.3%, and in the ISP, such cells were not observed at all. Cells simultaneously immunoreactive to ZnT3 and GAL comprised 11.9% ± 1.5% of all ZnT3-LI non-cholinergic neurons in the MP, but such perikarya were not noted in the OSP, and in the ISP, they amounted to only 2.8% ± 0.3%. The degree of co-localisations of ZnT3 with VIP, SOM and SP was levelised in all “kinds” of plexuses and was rather slight, because they did not exceed 5% of all non-cholinergic ZnT3-LI cells regardless of the “kind” of plexus. An especially low level of co-localisation of ZnT3 with LENK was observed. Cells simultaneously immunopositive to ZnT3 and LENK were observed only in the MP, and the percentage of these amounted to barely 0.5% ± 0.2% of all non-cholinergic ZnT3^+^ neurons. During the present study, the co-localisations of ZnT3 and NPY, as well as ZnT3 and CGRP, were not observed in non-cholinergic ZnT3-LI cells in any plexus of the ileal enteric nervous system under physiological conditions.

A completely different situation was observed in the ISP. In this plexus, a much higher percentage of cholinergic ZnT3-LI neurons were immunoreactive to SOM, VIP and/or SP. These values amounted to 24.1% ± 1.2%, 33.0% ± 3.6% and 55.1% ± 1.3%, respectively (Table 1). Moreover, 65.2% ± 1.2% of cholinergic ZnT3^+^ cells in the ISP were also immunoreactive to GAL, contrary to MP and OSP, where ZnT3^+^/VAChT^+^/GAL^+^ neurons were not observed at all. During the present study, immunoreactivity to nNOS, leu-enkephalin (LENK), neuropeptide Y (NPY) and calcitonin gene-related peptide (CGRP) was not observed in cholinergic ZnT3-positive neurons in any enteric plexus.

### 2.2. ZnT3 in the ENS during the Inflammatory Process

Experimentally induced colitis changed both the percentage of ZnT3-positive cells and the degree of co-localisation of ZnT3 with the other active factors studied (Table 1). The observed modifications clearly depended on the “kind” of enteric plexus, as well as the type of neurochemical substance.

During the inflammatory process, an evident increase in the percentage of ZnT3^+^ neurons (in relation to all protein gene product (PGP 9.5)-positive cells) was observed within all enteric plexuses. In the MP and OSP, these values were approx. two-fold higher than in the control group and amounted to 84.1% ± 3.9% and 85.6% ± 2.0%, respectively. Changes in the ISP were less pronounced, but also clearly visible (an increase from 48.6% ± 4.8% to 79.0% ± 3.2%).

#### Changes in Neurochemical Characterisation of ZnT3^+^ Enteric Neurons during Inflammation

##### Myenteric Plexus

Inflammation influenced the neurochemical characterisation of both cholinergic and non-cholinergic enteric neurons immunoreactive to ZnT3. In the MP (Figure 1, Table 1), an increase of immunoreactivity in the majority of substances studied was observed in both classes of neurons, and the magnitude of these changes was most visible in non-cholinergic (VAChT^−^) cells. The highest percentage of ZnT3^+^/VAChT^−^ neuronal cells during inflammation was also immunopositive to nNOS (57.0% ± 4.0%), VIP (52.1% ± 2.8%) and/or LENK (41.2% ± 2.7%). ZnT3-LI cholinergic cells (VAChT^+^) contained only VIP (11.2% ± 1.9%), GAL (5.4% ± 1.5%) and SP (4.2% ± 1.1%). Contrary to the majority of substances studied, the number of ZnT3-positive cells (both cholinergic and non-cholinergic) immunoreactive to SOM within MP dropped to zero.

##### Outer Submucous Plexus

In the OSP (Figure 2), an increase in the percentage of cholinergic ZnT3-positive cells simultaneously immunoreactive to VIP (from 1.4% ± 0.8% to 3.8% ± 1.4%) and/or GAL (from 0% to 4.7% ± 0.9%) was noted, contrary to neurons ZnT3^+^/VAChT^+^/SOM^+^ and ZnT3^+^/VAChT^+^/SP^+^, where inflammation caused a decrease from 7.4% ± 1.4% to 1.1% ± 0.8% and 4.6% ± 1.6% to 2.8% ± 1.4%, respectively. Moreover, in the population of OSP non-cholinergic neurons (just as in the MP), the observed changes were most visible. The percentage of ZnT3^+^/VaChT^−^ neurons immunoreactive to the majority of substances studied was higher during inflammation. The highest number of ZnT3^+^ non-cholinergic cells was also immunopositive to SOM (43.0% ± 3.6%) and/or GAL (36.3% ± 7.5%) (Table 1).

##### Inner Submucous Plexus

During inflammation, the degree of co-localisation of ZnT3 with the majority of substances studied in cholinergic neurons within the ISP (Figure 3) was several times lower than in control animals. The inflammatory process caused a decrease in the percentage of neurons immunoreactive to ZnT3/VAChT/SOM (from 24.1% ± 1.2% to 4.1% ± 08%), ZnT3/VAChT/VIP (from 33.0% ± 3.6% to 7.3% ± 1.6%), ZnT3/VAChT/SP (from 55.1% ± 1.3% to 6.8% ± 1.5%) and ZnT3/VAChT/GAL (from 65.2% ± 1.2% to 4.7% ± 1.3%) (Table 1). Contrary to cholinergic neurons, an increase of the percentage of cells immunoreactive to the majority of all substances studied was observed in non-cholinergic ZnT3-positive perikarya. During inflammation, most of the ZnT3^+^/VAChT^−^ neurons were also immunopositive to GAL (54.7% ± 7.4%) and/or nNOS (51.4% ± 3.5%) (Table 1).

## 3. Discussion

The results obtained during the present study show that ZnT3-positive neurons are relatively numerous in the ENS of the porcine ileum, which is in accordance with previous studies on humans [10] and other parts of the porcine digestive tract [19,20]. In terms of the numerical account of ZnT3-positive neurons, the ENS is very different from other previously studied parts of the peripheral autonomic nervous system, where such perikarya were rather sparse [14]. These observations strongly suggest the important role of ZnT3 within enteric neurons, but the factors that affect the expression of this peptide in enteric neurons under physiological conditions, as well as the exact functions of ZnT3 in the GI tract, are unknown. During the present study, all ZnT3-LI cells observed in the enteric plexuses were also immunoreactive to PGP 9.5. This fact shows that ZnT3 in the ENS is present solely in neuronal cells, which is in agreement with previous studies [10,19,20]. On the other hand, ZnT3-positive cells scattered in the intestinal wall outside of the enteric plexuses were not neurons (PGP 9.5-negative). This observation confirms previous studies describing the presence of ZnT3 in pancreatic β cells [33]. Presently, ZnT3-LI non-neuronal cells have not been observed in the intestine, and the character and functions of such cells are completely unknown.

One of the more interesting aspects of the occurrence of ZnT3 in the GI tract is the dependence of its level on the amount of zinc in food. Previous studies showed that a diet deficient in zinc caused changes in mRNA levels for various ZnT family peptides in the pancreas, digestive tract and central nervous system of rodents [34,35,36], but at present, the influence of food on ZnT3 in the ENS has not been studied. The role of this zinc transporter in the GI tract also remains unknown, and all assumptions concerning this problem are based on an analogy with the central nervous system, where the functions of ZnT3 are better known. Namely, this zinc transporter could take part in the conduction of sensory stimuli and regulate neuronal secretion [37,38]. Moreover, ZnT3 is regarded as a marker of zinc-enriched terminals, which are associated with inhibitory processes [16]. The present study, in which many other neuroactive substances were observed in ZnT3^+^ neurons, suggests that this zinc transporter is present in varied classes of enteric neurons and, consequently, could play various roles not only in the central nervous system, but also in the ENS. Proof of this seems to be the presence of ZnT3 in both cholinergic and non-cholinergic nNOS^+^ enteric neurons, observed during the present study. Acetylcholine, the main neuromediator of the ENS, present in different functional classes of enteric neurons [2], is responsible for the activation of intestinal motility [5,6] and the secretory functions of the GI tract [39]. In contrast, nitric oxide (NO), which is characteristic mainly for non-cholinergic neuronal cells, may act as an inhibitory factor. It is known that NO has a relaxatory effect on intestinal muscles, suppressing the exudation of electrolytes and hormones by the GI tract and also regulating blood flow in the gut by blood vessel relaxation [40,41,42]. Moreover, differences in neurochemical characterisation between ZnT3-positive enteric neurons in the ileum (this study) and the jejunum [19] or duodenum [20] may suggest that the exact functions of ZnT3 in the ENS depend on the fragment of the digestive tract.

The influence of experimental colitis on ZnT3-positive enteric neurons has been observed during the present study. These observations confirm the relatively well-known capabilities of the ENS to changes under various pathological factors [26] and strongly suggest that ZnT3 can take part in adaptive, neuroprotective and/or regenerative reactions within the GI tract. Previous studies on the central nervous system described the increase in mRNA levels for various ZnT family peptides under cerebral ischemia [43], which is probably connected with the augmentation of the amount of zinc in neuronal tissue during this type of pathological process [44,45]. It should be pointed out that zinc in non-physiological conditions can act on neurons in two ways. On the one hand, zinc exhibits neuroprotective activity and reduces neuronal damage [46], but on the other hand, it is known that this metal can be deposited in the cytoplasm and present neurotoxic effects [44]. In this case, the increase in the expression of ZnT transporters is intended to protect neuronal cells. Similar mechanisms probably occur in the GI tract and are the basis of the changes observed during the present study. This is all the more likely as the neuroprotective role of ZnT3 has been described in the central nervous system during Alzheimer’s disease [47], and interactions between ZnT transporters and NO in neuroprotective activity have also been observed. The high degree of co-localisation of ZnT3 with nNOS, noted in the present study especially during inflammation, seems to confirm these interactions. Moreover, other substances (VIP, GAL) that co-localised with ZnT3^+^ enteric neurons observed during this experiment are known as factors that have neuroprotective properties [48,49,50].

Another argument for the neuroprotective role of ZnT3 in the ENS is the fact that pathological processes cause an increase in the expression of active factors that exhibit neuroprotective effects with a simultaneous decline in the amount of other substances [49]. This situation has been observed in the present study. Namely, a decrease in the percentage of ZnT3-positive cholinergic neurons has been noted, and acetylcholine is the main neuromediator in the ENS under physiological conditions [5,6]. At the same time, an increase in the degree of co-localisation of ZnT3 with other substances, such as nNOS, VIP, SP, SOM, LENK and/or GAL, has generally been observed.

The co-localisation of ZnT3 with the abovementioned neurochemical factors suggests that this zinc transporter can play similar roles (besides neuroprotective) for these neurochemical factors. ZnT3 are possibly inhibitory factors in the ENS, like VIP or nNOS [40,41,51], and can also take part in the conduction of sensitive stimuli, like SP or LENK [9,52,53]. These functions of ZnT3 have also been described in the central nervous system [16].

On the other hand, it should be pointed out that the functions of ZnT3 in the central nervous system, although important (by analogy) to explain its role in the ENS, do not clarify all doubts. For example, ZnT3-positive nerve fibres, known as zinc-enriched nerves, which can play an inhibitory role, have been studied in the central nervous system [16], where both the present experiment and previous studies [10,19,20] did not reveal nerve fibres to be immunoreactive to this zinc transporter in the intestinal wall. Moreover, the exact reasons for changes in immunoreactivity to ZnT3 observed during the present study are difficult to explain, as they may arise from modifications in various stages of peptide synthesis, such as transcription or translation, as well as during post-translational modifications and even shifts in the transport of ZnT3 within neuronal cells.

The changes observed during the present investigations are most likely related to disturbances in zinc homeostasis. It is relatively well established that zinc, as one of the essential micronutrients, plays important roles during diseases localised in various organs and systems. Previous studies showed that the deregulation of zinc homeostasis can be involved in processes connected with Alzheimer’s disease, asthma and diabetes [47,54,55], as well as pathological processes within the GI tract, including inflammatory bowel disease, ulcerative colitis and Crohn’s disease [56,57].

On the other hand, zinc transporters are key factors implicated in the regulation of zinc distribution in a living organism. There are two main families of zinc transporters: the ZnT proteins (SLC30) and the Zip (Zrt- and Irt-like proteins) family (solute-linked carrier 39—SLC39) [58]. The first of these transport zinc ions from the cytoplasm outside of cells or into the intracellular vesicle, thereby reducing intracellular zinc concentration. The second group has the opposite action, as they promote zinc transport from extracellular space into the cytoplasm.

ZnT3, which is mainly localised in neuronal cells, transports zinc ions from the cytoplasm into synaptic vesicles [12], and the correlation between the expression of this zinc transporter and synaptic Zn^2+^ levels has been described [12,59].

The exact roles in the maintenance of synaptic zinc ions homeostasis in the ENS remain unknown, but they are probably similar to those observed within the CNS. It is known that synaptic Zn^2+^ in the brain and spinal cord plays a modulatory role in synaptic transmission, and its homeostasis determines the correct functioning of the nervous system [59]. Both synaptic Zn^2+^ deficiency and excess causes disturbances in postsynaptic neurons. In the first case, dysfunctions of postsynaptic neurons have been observed, and in the second neurodegeneration has been observed [59,60]. Given this context, Zn^2+^ is regarded as a potentially neurotoxic factor that is involved in neuronal loss and plays some role in neurodegenerative diseases, including Huntington’s disease, Parkinson’s disease and amyotrophic lateral sclerosis [60]. On the other hand, it is known that both ZnT3 and synaptic Zn^2+^ are inherent to correct brain development [61,62].

The increase of ZnT3-like immunoreactivity observed in the present study was probably connected with synaptic zinc ions excess. This effect could be due to the direct neurodegenerative influence of the inflammatory process. On the other hand, it has been connected with neuroprotective and/or adaptive processes in enteric neurons. Namely, inflammation may have been responsible for the dysfunction of postsynaptic neurons, which is often connected with a deficiency of synaptic Zn^2+^ [59]. For this reason, presynaptic neurons enhanced the expression of ZnT3 in order to maintain zinc homeostasis. The increase in ZnT3-like immunoreactivity could also be the result of an excessive concentration of intracellular cytoplasmic zinc. This is more likely as previous studies described the activation of ZIP transporters (responsible for the transport of zinc ions from extracellular fluid into the cytoplasm) during inflammatory processes [63].

The observed changes could also be connected with other functions of zinc in the intestine, including participation in the maintenance of intestinal mucosal layer integrity, controlling leukocyte infiltration, as well as excretion of pro-inflammatory factors [64,65,66]. Moreover, it is known that ZnT3 probably takes part in sensory stimuli conduction [67], and fluctuations in this transporter’s levels observed during the present study were caused by pain processes during inflammation.

## 4. Materials and Methods

The present study was performed on 10 immature female pigs of the Large White Polish breed at the age of approximately eight weeks. The animals were kept under standard laboratory conditions. All actions connected with the experiment were carried out in compliance with the regulations of the Local Ethical Committee in Olsztyn (Poland)—(decision numbers: 90/2007 from 20 November 2007—submission 90/2007/N and 27/2009 from 18 March 2009—submission 26/2009/DTN).

Sows were randomly divided into two experimental groups: control (C group; *n* = 5) without any surgical operations, and animals in which experimental acute colitis and visceral pain were induced according to the method described previously by Miampamba et al. [68] and modified by Gonkowski et al. [69] (Inflammatory—I group; *n* = 5).

The pigs of I group were subjected to the following experimental procedures: (a) premedication with Stressnil (Janssen, Beerse, Belgium, 75 µL/kg of body weight given intravenously) 15 min before the application of the main anaesthetic; (b) general anaesthesia using sodium thiopental (Thiopental, Sandoz, Kundl-Rakúsko, Austria; 20 mg/kg of body weight given intravenously); and (c) median laparotomy. During laparotomy, the sows were injected with 80 µL of 10% formalin solution (microinjections of 5–8 µL) into the wall of the ileum five centimetres before the ileocecal valve. The group of “sham” operated animals (injections of saline solution instead of formalin) was deliberately abandoned during the experiment due to the fact that previous studies clearly showed that this type of manipulation of the intestine does not influence the neurochemical coding of enteric neurons [9,27,70]. Reducing the number of animals was in accordance with the regulations of the Local Ethical Committee.

After five days, all animals (C and I groups) were euthanized by an overdose of sodium thiopental and then perfused transcardially with 4% buffered paraformaldehyde (pH 7.4) prepared ex tempore.

The same parts (about 2 cm long) of the ileum (from the area where injections of the formalin solution were made and inflammatory changes were observed in animals of I group) were collected and post-fixed by immersion in the same solution used during transcardial perfusion for 20 min. Then tissues were rinsed in a phosphate buffer (0.1 M; pH 7.4; 4 °C) for three days with the buffer changing every day and, finally, stored in 18% sucrose (at least 10 days). After this period, the fragments of ileum were frozen at −25 °C and cut into 10 µm-thick cryostat sections, which were subjected to standard triple-labelling immunofluorescence according to the method described previously by Wojtkiewicz et al. [19]. In short, the immunofluorescence technique was performed as follows: slices with tissue sections were air-dried at room temperature (rt) for 45 min and incubated with a blocking solution (10% normal goat serum, 0.1% bovine serum albumin, 0.01% NaN_3_, Triton x-100 and thimerozal in PBS) for 1 h (rt). After this period, tissues were incubated overnight in a humid chamber (rt) with a mixture of three primary antibodies: (1) anti-protein gene product 9.5 (PGP 9.5—used here as a pan-neuronal marker); (2) anti-vesicular acetylcholine transporter (VAChT—used here as a marker of cholinergic neurons); and (3) directed against one of the following substances: calcitonin gene-related peptide (CGRP), galanin (GAL), leu-enkephalin (LENK), neuronal isoform of nitric oxide synthase (Nnos—used here as a marker of nitrergic neurons), neuropeptide Y (NPY), somatostatin (SOM), substance P (SP) and vasoactive intestinal polypeptide (VIP). The following day, slices were incubated (1 h, rt) with species-specific secondary antisera conjugated to 7-amino-4-methylcoumarin-3-acetic acid (AMCA), fluorescein isothiocyanate (FITC) or biotin, which was then visualised by a streptavidin-CY3 complex (1 h; rt). The specification of primary and secondary antibodies used in the present study is shown in Table 2. Each stage of immunofluorescence labelling was followed by rinsing the slices with PBS (3 × 10 min, pH 7.4). The specificity of antisera was tested by standard controls, including pre-absorption of antibodies with the appropriate antigen, omission test and replacement of antisera by non-immune sera. The abovementioned controls completely eliminated labelling in the tissue.

The overall percentage of ZnT3-LI neurons was defined by the examination of at least 700 PGP-9.5-labelled cell bodies for ZnT3-like immunoreactivity in each “kind” of enteric plexus (i.e., muscular, outer submucous and inner submucous plexuses), and the number of neurons immunoreactive to PGP 9.5 was treated as 100%. In the case of the study on the co-localisation of ZnT3 with acetylcholine, at least 300 ZnT3-positive cell bodies in particular types of enteric ganglia were examined for immunoreactivity to VAChT. In these studies, ZnT3-positive neurons were considered as representing 100%. To determine the chemical coding of cholinergic and non-cholinergic neurons immunoreactive to ZnT3, at least 300 ZnT3^+^/VAChT^+^ and ZnT3^+^/VAChT^−^ were studied for immunoreactivity against particular substances. Evaluation of immunopositive cells and the counting of neurons were performed by two independent investigators. During the present study, only double- or triple-labelled neuronal cell bodies with a clearly visible nucleus were determined under an Olympus BX51 microscope equipped with epi-fluorescence and appropriate filter sets, pooled and presented as mean ± SEM. The section of ileum that was the subject of the study was located at least 100 µm apart to prevent double counting of neurons. All pictures were captured with a digital camera connected to a PC. Statistical analysis was performed with the Anova test using Statistica 10 software (StatSoft Inc., Tulsa, OK, USA). The differences were considered statistically significant at *p* ≤ 0.05.

## 5. Conclusions

In summary, the present investigation shows that ZnT3 is present in relatively numerous populations of enteric neurons in the porcine ileum, both under physiological conditions and during experimental inflammation in cholinergic and non-cholinergic cells. The wide range of active substances (including SP, VIP, SOM, nNOS and GAL) that co-localise with ZnT3 suggests that this zinc transporter occurs in various classes of enteric neurons and may take part in different regulatory processes within the intestinal wall. Meanwhile, changes in ZnT3-like immunoreactivity during experimental inflammation could denote the functions of ZnT3 in adaptive and/or regenerative reactions in the ENS. ZnT3 is probably present in enteric neurons, which use zinc ions as a neuromodulator, and fluctuations in ZnT3 expression are connected with disturbances in zinc ions homeostasis. Moreover, it is known that, on the one hand, zinc is essential for correct development and functioning of the ENS [25], and during inflammation or nerve damage, ZnT3 can play a neuroprotective role. On the other hand, zinc ions can exhibit neurodegenerative activity [59]. Therefore, a lot of aspects connected with the role of ZnT3 within the ENS remain unknown and require further investigations.

## Figures and Tables

**Table 1:** Zinc transporter 3-like immunoreactive (ZnT3-LI) neurons in the enteric nervous system (ENS) of the porcine ileum under physiological conditions (C group) and during chemically-induced inflammation (I group).

Enteric Plexus	MP		OSP		ISP	
	C Group	I Group	C Group	I Group	C Group	I Group
**PGP^+^/ZnT3^+ 1^**	**42.3 ± 4.7**	**84 ± 3.9**	**43.5 ± 6.8**	85.6 ± 2.0	**48.6 ± 4.8**	79.0 ± 3.2
**ZnT3^+^/VAChT^+ 2^**	**85.0 ± 5.8**	**41.0 ± 2.7**	**82.0 ± 5.9**	31.3 ± 2.8	**90.0 ± 7.2**	34.0 ± 3.0
ZnT3^+^/VAChT^+^/CGRP^+ 3^	0	0	0	0	0	0
ZnT3^+^/VAChT^+^/GAL^+ 3^	0	5.4 ± 1.5	0	4.7 ± 0.9	65.2 ± 1.2	4.7 ± 1.3
ZnT3^+^/VAChT^+^/LENK^+ 3^	0	0	0	0	0	0
ZnT3^+^/VAChT^+^/NOS^+ 3^	0	0	0	0	0	0
ZnT3^+^/VAChT^+^/NPY^+ 3^	0	0	0	0	0	0
ZnT3^+^/VAChT^+^/SOM^+ 3^	2.9 ± 1.4	0	7.4 ± 1.4	1.1 ± 0.8	24.1 ± 1.2	4.1 ± 0.8
ZnT3^+^/VAChT^+^/SP^+ 3^	1.2 ± 0.5	4.2 ± 1.2	4.6 ± 1.6	2.8 ± 1.4	55.1 ± 1.3	6.8 ± 1.5
ZnT3^+^/VAChT^+^/VIP^+ 3^	2.9 ± 1.4	11.2 ± 1.9	1.4 ± 0.8	3.8 ± 1.4	33.0 ± 3.6	7.3 ± 1.6
**ZnT3^+^/VAChT^− 2^**	**15.0 ± 5.6**	59.0 ± 2.7	**18.0 ± 5.9**	68.7 ± 2.8	**10.0 ± 7.2**	**66.0 ± 3.0**
ZnT3^+^/VAChT^−^/CGRP^+ 3^	0	0	0	0	0	0
ZnT3^+^/VAChT^−^/GAL^+ 3^	11.9 ± 1.5	30.2 ± 3.4	0	36.3 ± 7.5	2.8 ± 0.1	54.7 ± 7.4
ZnT3^+^/VAChT^−^/LENK^+ 3^	0.5 ± 0.2	41.2 ± 2.7	0	0	0	0
ZnT3^+^/VAChT^−^/NOS^+ 3^	17.1 ± 1.1	57.0 ± 4.0	7.4 ± 1.3	10.3 ± 2.4	0	0
ZnT3^+^/VAChT^−^/NPY^+ 3^	0	0	0	0	0	0
ZnT3^+^/VAChT^−^/SOM^+ 3^	3.4 ± 1.3	0	1.4 ± 0.6	43.0 ± 3.6	3.7 ± 1.0	38.6 ± 1.5
ZnT3^+^/VAChT^−^/SP^+ 3^	1.1 ± 0.6	32.2 ± 0.4	4.7 ± 1.8	9.5 ± 2.4	4.1 ± 1.2	36.9 ± 1.5
ZnT3^+^/VAChT^−^/VIP^+ 3^	3.9 ± 0.3	52.1 ± 2.8	4.6 ± 1.6	20.7 ± 9.7	2.9 ± 1.3	51.4 ± 3.5

MP: myenteric plexus; OSP: outer submucous plexus; ISP: inner submucous plexus; ^1^ The relative frequency of ZnT3-LI neuronal cells is presented as % (mean ± SEM) in relation to all neurons counted within the enteric ganglionated plexuses stained for PGP 9.5 (PGP 9.5-LI cells were treated as 100%); ^2^ The percentage of VAChT^+^ or VAChT^−^ neurons is presented as % (mean ± SEM) of all neurons counted within the ganglionated plexuses stained for ZnT3; ^3^ The relative frequency of neurons immunoreactive to particular substances is presented as % (mean ± SEM) of all ZnT3^+^/VAChT^+^ or ZnT3^+^/VAChT^−^ neurons counted within the ganglionated plexuses. Statistically significant data (*p* ≤ 0.05) are marked by a different colour (red—increase, green—decrease of the percentage of a particular neuronal population in I group compared to C group).The main groups of cells (general percentage of ZnT3^+^, cholinergic ZnT3^+^ and non-cholinergic ZnT3^+^ neurons) are indicated with bold font.

**Table 2:** Specification of immune reagents used in the study: PGP9.5—protein gene product 9.5, ZnT3—zinc transporter 3, NOS—nitric oxide synthase, VIP—vasoactive intestinal peptide, SOM—somatostatin, VAChT—Vesicular acetylcholine transporter, NPY—neuropeptide Y, GAL—galanin, CGRP—calcitonin-gene related peptide, FITC—fluorescein isothiocyanate, AMCA—7-amino-4-methylcoumarin-3-acetic acid, H heavy chain, L light chain.

**Primary Antibody**				
**Antisera**	**Code**	**Host Species**	**Dilution**	**Supplier**
PGP9.5	7863-2004	Mouse	1:2000	Biogenesis Inc., Poole, UK; www.biogenesis.co.uk
ZnT3	-	Rabbit	1:600	Gift from prof. Palmiter, University of Washington Seattle, WA, USA
NOS	N2280	Mouse	1: 2000	Sigma-Aldrich, Saint Louis, MS, USA; www.sigma-aldrich.com
VIP	9535-0504	Mouse	1: 2000	Biogenesis Inc.
SP	8450-0505	Rat	1:300	Biogenesis Inc.
SOM	8330-0009	Rat	1: 100	Biogenesis Inc.
LENK	4140-0355	Mouse	1: 1000	Biogenesis Inc.
VAChT	H-V007	Goat	1: 2000	Phoenix, Pharmaceuticals, INC., Belmont, CA, USA; www.phoenixpeptide.com
NPY	NZ1115	Rat	1:300	Biomol Research Laboratories Inc., Plymouth, PA, USA
GAL	T-5036	Guinea pig	1:1000	Peninsula Labs., San Carlos, CA, USA; see Bachem AG; www.bachem.com
CGRP	T-5027	Guinea pig	1:1000	Peninsula Labs.
**Secondary Antibodies**				
**Reagent**			**Dilution**	**Supplier**
FITC-conjugated donkey-anti-mouse IgG (H+L)			1:800	Jackson, 715-095-151, West Grove, PA, USA
FITC-conjugated donkey-anti-rat IgG (H+L)			1:800	Jackson, 712-095-153
FITC-conjugated donkey-anti-guinea pig IgG (H+L)			1:1000	Jackson, 706-095-148
FITC-conjugated donkey-anti-goat IgG (H+L)			1:1000	Jackson, 705-096-147
Biotinylated goat anti-rabbit immunoglobulins			1:1000	DAKO, E 0432, Carpinteria, CA, USA
Biotin conjugated F(ab)’ fragment of affinity Purified anti-rabbit IgG (H+L)			1:1000	BioTrend, 711-1622, Cologne, Germany
AMCA-conjugated donkey-anti-mouse IgG (H+L)			1:50	Jackson, 715-155-151
AMCA-conjugated donkey-anti-rat IgG (H+L)			1:50	Jackson, 715-155-153
AMCA-conjugated donkey-anti-goat IgG (H+L)			1:50	Jackson, 705-156-147
CY3-conjugated Streptavidin			1:9000	Jackson, 016-160-084
